# Exon-primed intron-crossing (EPIC) markers for non-model teleost fishes

**DOI:** 10.1186/1471-2148-10-90

**Published:** 2010-03-31

**Authors:** Chenhong Li, Jean-Jack M Riethoven, Lingbo Ma

**Affiliations:** 1 School of Biological Sciences, University of Nebraska - Lincoln, NE 68588-0118, USA; 2 Bioinformatics Core Research Facility, University of Nebraska - Lincoln, NE 68588-0665, USA; 3 East China Sea Fisheries Research Institute, Chinese Academy of Fisheries Sciences, Shanghai, 200090, PR China

## Abstract

**Background:**

Exon-primed intron-crossing (EPIC) markers have three advantages over anonymous genomic sequences in studying evolution of natural populations. First, the universal primers designed in exon regions can be applied across a broad taxonomic range. Second, the homology of EPIC-amplified sequences can be easily determined by comparing either their exon or intron portion depending on the genetic distance between the taxa. Third, having both the exon and intron fragments could help in examining genetic variation at the intraspecific and interspecific level simultaneously, particularly helpful when studying species complex. However, the paucity of EPIC markers has hindered multilocus studies using nuclear gene sequences, particularly in teleost fishes.

**Results:**

We introduce a bioinformatics pipeline for developing EPIC markers by comparing the whole genome sequences between two or more species. By applying this approach on five teleost fishes whose genomes were available in the Ensembl database http://www.ensembl.org, we identified 210 EPIC markers that have single-copy and conserved exon regions with identity greater than 85% among the five teleost fishes. We tested 12 randomly chosen EPIC markers in nine teleost species having a wide phylogenetic range. The success rate of amplifying and sequencing those markers varied from 44% to 100% in different species. We analyzed the exon sequences of the 12 EPIC markers from 13 teleosts. The resulting phylogeny contains many traditionally well-supported clades, indicating the usefulness of the exon portion of EPIC markers in reconstructing species phylogeny, in addition to the value of the intron portion of EPIC markers in interrogating the population history.

**Conclusions:**

This study illustrated an effective approach to develop EPIC markers in a taxonomic group, where two or more genome sequences are available. The markers identified could be amplified across a broad taxonomic range of teleost fishes. The phylogenetic utility of individual markers varied according to intron size and amplifiability. The bioinformatics pipelines developed are readily adapted to other taxonomic groups.

## Background

Molecular studies aimed at understanding species limits and population dynamics, are often thwarted by conflicting results arising from different markers. Although mitochondrial genes are frequently used for detecting genetic patterns in recently diverged populations [1], multiple independent nuclear loci are generally thought to provide more reliable estimates of the evolutionary history of populations [2]. Not only do multilocus analyses provide better estimates of divergence times and effective population sizes [3], but they also provide more reliable estimates of species trees, one of the primary goals of systematics. Gene trees are often affected by factors other than divergence, such as incomplete lineage sorting and migration. The most widely accepted way to sort out the historical signals from stochastic effects of gene trees is to extract the common patterns from many independent loci. In the same vein, in the state of the art approaches for studying species delimitation and population dynamics, such as coalescence based methods [4,5] and individual assignment tests [6,7], have always emphasized using more loci.

One class of markers commonly used in such studies are anonymous nuclear loci, For example, they have been used to study demography of eastern fence lizard [8] and statistical phylogeography of bird [3]. The major drawback of such approaches is the effort that has to be invested in developing the markers, which usually involves extensive cloning and sequencing of genomic DNA. In addition, the markers developed for one taxon often cannot be applied to other taxa, due to the high mutation rates in priming sites.

An alternative to anonymous nuclear markers is intron sequence. Introns have been successfully used in species-level studies [9-12]. The common strategy to sequence introns is to design primers on adjacent exon regions and amplify across the intron, so called exon-primed intron-crossing (EPIC) markers [13-15]. Because exons are usually more conserved than introns and most anonymous loci, the EPIC primers can generally be applied across a wider taxonomic range of organisms. An further advantage of EPIC markers is that having both the exon and intron fragments can be useful for examining genetic variation at the intraspecific and interspecific level simultaneously, a feature that is particularly useful when studying species complexes. Having both the exon and intron sequences also helps in assessing the orthology of collected sequences [16].

Development of molecular markers has benefited from the growth of publicly accessible genomes and EST data sets. A few bioinformatics tools have been successfully used to explore the potential of intron markers in plants [17,18]. Recently, Backström [19] developed intron markers for a non-model species, zebra finch (*Taeniopygia guttata*), by comparing its expressed sequence tag (EST) sequences with the genome sequences of chicken. The success of this study demonstrates that genomic data from a model organism can be used effectively to develop EPIC markers for non-model species. These resources "pave the way for easy multilocus study of evolving populations and lineages of birds, and bring the goal of quickly turning nonmodel species into ecological genomic models tantalizingly close" [20].

The development and use of introns in fish studies, however, are still sporadic [11,12,21,22] and few automated bioinformatics tools have been developed. On the other hand, the genomic resources of fishes are much more extensive than are those of birds. Currently, there are five full genome sequences of fishes (*Danio rerio, Oryzias latipes, Gasterosteus aculeatus, Takifugu rubripes *and *Tetraodon nigroviridis*) curated at the Ensembl genome browser http://www.ensembl.org. These five fishes encompass a wide phylogenetic breadth from the ostariophysans to tetraodontiforms. In addition, there are many EST sequences for other fish species available http://www.ncbi.nlm.nih.gov. Using these published fish genomes, Li et al. [23] have developed a bioinformatics pipeline to identify single-copy and conserved exons for phylogenetics. The strategy they used can be adapted to search for intron markers flanked by single-copy and conserved exons. The objectives for our particular study were: (1) to develop a bioinformatics tool to search for intron markers flanked by single-copy conserved exons; (2) to identify such markers for teleosts fish using the five published fish genomes; (3) to design primers and survey a set of teleost fish using the markers identified with our pipeline; (4) to investigate the relationship between intron size, amplifiability and genetic distance of target taxa in order to predict how distantly related two taxa can be and still amplify for the same markers.

## Results

### The bioinformatics tool and EPIC markers developed

We wrote Perl scripts to automate the pipelines (available upon request from C. Li). A well-annotated primary genome is required as the query while one or more reference genomes are needed as the subjects. We used the genome of *D. rerio *as the query and the genomes of *O. latipes, G. aculeatus, T. rubripes *and *T. nigroviridis *as the references. It took 26 hours and 34 minutes CPU time to complete the runs on a single core of a Dell PowerEdge 1955 quad core E5345 machine. There were 137,640 large (≥ 100 bp) coding sequences (CDS) found in *D. rerio*, from which 62,856 were identified as "single-copy" (no other sequences having more than 20% coverage and more than 40% similar to itself). After comparison with the other four genomes and screening by the intron size (≤ 1,000 bp in at least one species), 5051 EPIC markers were identified whose average identity in the flanking exon regions was larger than 65% among the five model species. When the required average identity of exons was increased to 80%, 2021 EPIC markers were found. When average identity of exons was increased to 85%, 210 EPIC markers were found (for the description of these markers see Table 1 and Additional file 1). From our previous experience in amplifying exon sequences across a wide range of taxa, we found that the markers worked well if the average identity was larger than 80%. So, potentially we could have thousands of useable candidate EPIC markers across teleost fishes. The parameter settings of the Perl scripts, such as the maximum intron length, identity and coverage are interactive; thus, can be adjusted for each study by the user. The bioinformatics tool developed in this study also was applied in identifying EPIC markers for chondrichthyans, using human as the query and a low 1.4× coverage genome of a chimaera (*Callorhinchus milii*) as the reference [24]. We used chimaera and human because chimaera is the only chondrichthyan with genome sequence available and human is the best-annotated vertebrate genome. Five hundred candidate EPIC markers were found from this comparison (unpublished data). This is quite a remarkable result given the poor coverage and quality of the chimaera genome data and its evolutionary distance to human as a comparison.

**Table 1:** The gene description, chromosomal location and start position of EPIC markers identified in this study.

Locus	Chromosome	Marker start (bp)	Gene description
59107E2	4	572793	UPF0027 protein C22orf28 homolog. [Source:Uniprot/SWISSPROT;Acc:Q6NZS4]
55378E1	Zv7_NA122	96106	Peroxisome proliferator activated receptor gamma coactivator 1 alpha (Fragment). [Source:Uniprot/SPTREMBL;Acc:Q52MY8]
55305E1	13	26342049	ret proto-oncogene [Source:RefSeq peptide;Acc:NP_858048]
40245E5	1	2590224	hypothetical protein LOC569455 [Source:RefSeq peptide;Acc:NP_001139076]
36298E1	7	18108484	hypothetical protein LOC415169 [Source:RefSeq_peptide;Acc:NP_001002079]
25073E1	4	1723855	60S ribosomal protein L18a [Source:UniProtKB/Swiss- Prot;Acc:Q7ZWJ4]
19231E4	21	10663833	spectrin alpha 2 [Source:RefSeq peptide;Acc:NP_001091958]
14867E1	9	33724200	60S ribosomal protein L8 [Source:UniProtKB/Swiss- Prot;Acc:Q6P0V6]
08680E3	19	45181216	karyopherin (importin) beta 1 [Source:RefSeq_peptide;Acc:NP_001032791]
08680E2	19	45180803	karyopherin (importin) beta 1 [Source:RefSeq_peptide;Acc:NP_001032791]
04174E20	25	13957143	CCR4-NOT transcription complex subunit 1 (CCR4- associated factor 1) [Source:UniProtKB/Swiss- Prot;Acc:A1A5H6]
01777E4	10	2555539	nucleoporin 155 [Source:RefSeq peptide;Acc:NP_956450]

Only the first 12 markers tested in this study are listed. For the other markers see Additional file 1. The markers are named using the last five numbers of the Ensembl gene name plus a sequential number to distinguish markers found in the same gene. The location information shown is from *D. rerio*.

**Figure 1:**
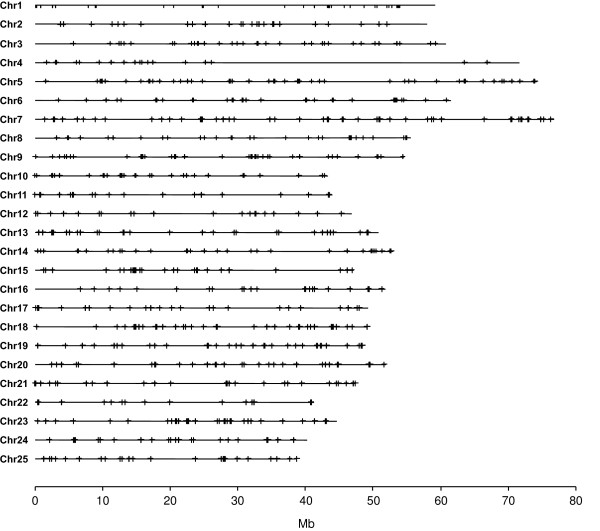
**The location of EPIC markers shown on the 25 chromosomes (Chr) of *Danio rerio***. The lines are chromosomes drawn to the scale (Mb). The crosses indicate the positions of EPIC markers. We show only the 2021 EPIC markers whose exonic portion has average identity larger than 80% among the five model fish species. Twelve of the 2021 EPIC markers with no chromosomal information are not included on this map.

The gene functions of the identified EPIC markers seem not to be restricted to any particular type (Table 1 and Additional file 1). The chromosomal positions of the 2021 EPIC markers with exon identity higher than 80% were mapped onto the 25 chromosomes of *D. rerio *(Figure 1), showing a good coverage of the genome.

### Intron size, amplifiability and the genetic distances between the target taxa

We randomly picked 12 EPIC markers from the 210 candidates to test the utility of these markers in teleosts (Table 1). One pair of primers was designed for each marker (Table 2). Primers were designed to have similar annealing temperatures, so that they would function under the similar PCR conditions. The success rate for amplifying these markers ranged from 44% to 100%, with 4174E20 amplified in all taxa and 8680E2 and 40245E5 only amplified in four of the nine taxa tested (Table 3). If we compare the success taxon-wise, both *D. rerio *and *Larimichthys crocea *worked for all 12 markers, while *Rhynogobius giurinus *only worked for five markers (Table 3).

**Table 2:** The primer sequences used for the 12 EPIC markers.

Locus	Forward primer sequence	Reverse primer sequence
59107E2	GGAGATGGGYGTGGACTGGTCYCT	ATTGTAGATCTCVTCCACCACCTGRAT
55378E1	ATGARGAAAATGAGGCCAACTTGCT	GCCACCTGKGTATTGATTATAGCTGAG
55305E1	CCTAGTGGACTGTARTAACGCCCCYCT	AAGCCATCCAGTTTGCATAAACACTATC
40245E5	CTGAGGAGGAYGGCTGGGARTTYGT	ACCATCAGCTTCACCACCTGCTC
36298E1	GATCCTGAGGGAYTCCCAYGGTGT	GGGCCAGGACTCTCYTGGTCTTGTAGT
25073E1	GTACTCTCKGTACATGTTGTGRGTKCC	GAAGGTGAARAACTTTGGBATCTGG
19231E4	CGGARGACTACGGACGTGATTTGAC	CTCCYTCCAGTGSTCCACAAACT
14867E1	CCACAARTACAAGGCCAAGAGRAACTG	GTTCTCCTTSTCCTGSACGGTCTT
8680E3	GGAGGAGARTTYAAGAAGTAYCTGGACAT	CSCCCTTCAGGCCCTGGATGAT
8680E2	GATATGGTGGAYTACCTGAACGASCTG	TCCTCAGCKATGTGGTGRATGAA
4174E20	CTYTCGCTGGCTTTGTCTCAAATCA	CTTTTACCATCKCCACTRAAATCCAC
1777E4	AGGAGYTGGTGAACCAGAGCAAAGC	AGATCRGCCTGAATSAGCCAGTT

**Table 3:** Taxa used and sequences collected in testing the 12 EPIC markers.

Species and higher classifications						Locus						
	
	1777E4	4174E20	8680E2	8680E3	14867E1	19231E4	25073E1	36298E1	40245E5	55305E1	55378E1	59107E2
**Clupeiformes**												
*Thryssa hamiltonii*		HM012491	HM012502	HM012498	HM012537	HM012550	HM012545		HM012506	HM012530		
**Cypriniformes**												
*Danio rerio*	HM012515	HM012492	HM012503	HM012499	HM012538	HM012551	HM012546	HM012565	HM012507	HM012531	HM012523	HM012558
*Hypophthalmichthys molitrix*	HM012512	HM012488		HM012495	HM012534	HM012547	HM012542	HM012562		HM012527	HM012520	HM012555
*Hypophthalmichthys nobilis*	HM012513	HM012489		HM012496	HM012535	HM012548	HM012543	HM012563		HM012528	HM012521	HM012556
**Tetraodontiformes**												
*Takifugu rubripes*	Ensembl	Ensembl	Ensembl	Ensembl	Ensembl	Ensembl	Ensembl	Ensembl	Ensembl	Ensembl	Ensembl	Ensembl
*Tetraodon nigroviridis*	Ensembl	Ensembl	Ensembl	Ensembl	Ensembl	Ensembl	Ensembl	Ensembl	Ensembl	Ensembl	Ensembl	Ensembl
**Gasterosteiformes**												
*Gasterosteus aculeatus*	Ensembl	Ensembl	Ensembl	Ensembl	Ensembl	Ensembl	Ensembl	Ensembl	Ensembl	Ensembl	Ensembl	Ensembl
**Beloniformes**												
*Oryzias latipes*	Ensembl	Ensembl	Ensembl	Ensembl	Ensembl	Ensembl	Ensembl	Ensembl	Ensembl	Ensembl	Ensembl	Ensembl
**Perciformes**												
*Larimichthys crocea*	HM012514	HM012490	HM012501	HM012497	HM012536	HM012549	HM012544	HM012564	HM012505	HM012529	HM012522	HM012557
*Siniperca chuatsi*	HM012511	HM012487		HM012493	HM012533		HM012541	HM012561	HM012504	HM012526	HM012519	
*Odontobutis potamophila*	HM012508	HM012484			HM012532		HM012539	HM012559		HM012524	HM012516	HM012552
*Micropercops swinhonis*	HM012509	HM012485		HM012494			HM012540	HM012560		HM012525	HM012517	HM012553
*Rhinogobius giurinus*	HM012510	HM012486	HM012500								HM012518	HM012554
	
Number of taxa amplified	8	9	4	7	7	5	8	7	4	8	8	7
Success rate	0.89	1.00	0.44	0.78	0.78	0.56	0.89	0.78	0.44	0.89	0.89	0.78

The sequences collected in this study are lodged in GenBank with accession numbers from HM012484 to HM012565. Ensembl means the sequence was retrieved from Ensembl database.

**Figure 2:**
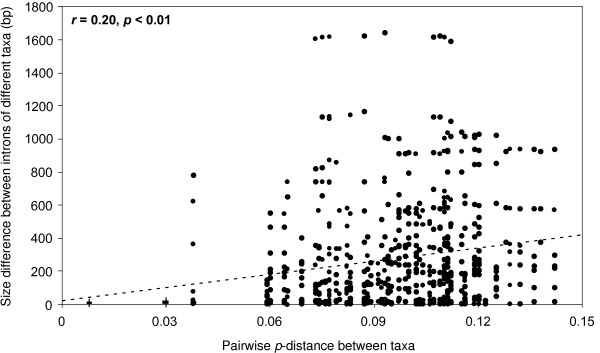
**The relationship between the p-distance and the difference in intron size among all taxa sequenced for the 12 EPIC markers (r = 0.20, p < 0.01)**.

At first glance, there appeared to be a positive relationship between variance in intron length and genetic *p*-distance among taxa (*r *= 0.20, *p*-distance; Figure 2). However, this relationship might be an artifact caused by the low variance in intron size when the *p*-distance was less than or equal to 0.03 and the generally higher difference in intron size when the *p*-distance was larger than 0.03 (Figure 2). In other words, closely related species had very similar intron size, whereas distantly related species (*p*-distance ≥ 0.03) generally had large difference in intron size; however, this difference did not continue to become larger with further increased genetic distance.

**Figure 3:**
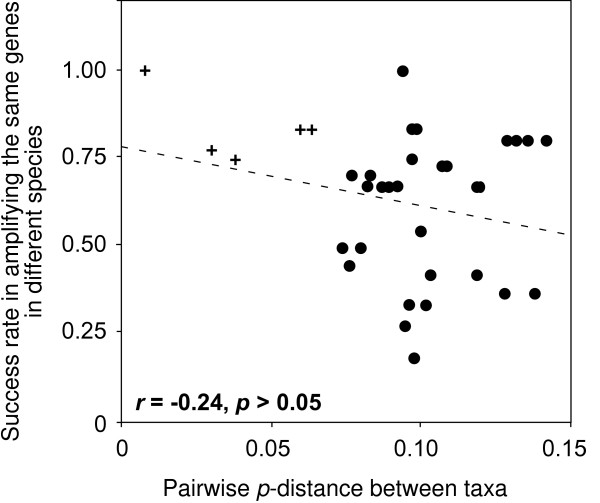
**The relationship between the p-distance and the success rate in amplifying the same gene among taxa**. The general trend is that the larger p-distance between target taxa, the smaller success rate in amplifying the same gene (r = -0.24, p = 0.15). However, confident assessment on the potential usefulness of a gene marker based on the experience in related taxa can only be made when their p-distance ≤ 0.06 in our study (the crosses).

If a suite of EPIC markers worked in one taxon, to predict whether we could use them in other taxa, we calculated the correlation between the amplifiability and the genetic *p*-distance between the taxa. A non-significant negative correlation was seen in our results (*r *= -0.24, *p *> 0.05; Figure 3). If the *p*-distance was less than or equal to 0.06 (the crosses in Figure 3), the success of amplifying EPIC in one taxon could be extended to the other taxa. But when the *p*-distance was larger than 0.06, the success in one taxon was not a good predictor for how the markers worked in the other taxa (Figure 3).

### Phylogenetic inference based on the exon portion of EPIC loci

**Figure 4:**
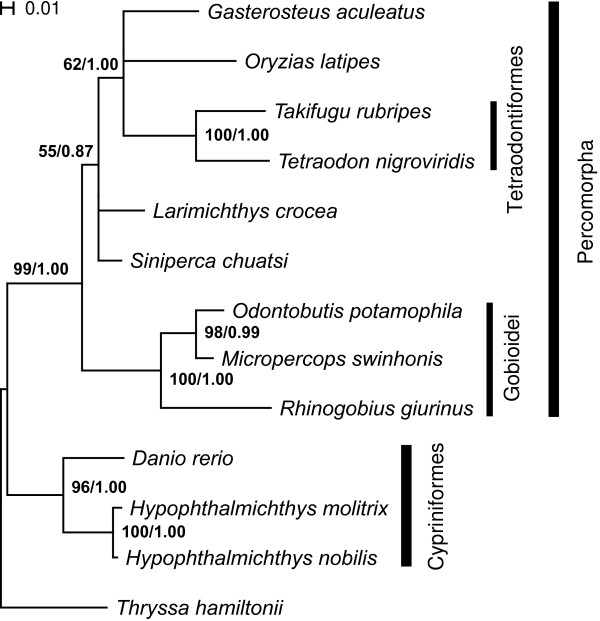
**Bayesian phylogeny of 13 taxa inferred from the exon parts of the 12 EPIC markers tested**. The ML analysis resulted in the same topology. The numbers by the branches are ML bootstrap values and Bayesian posterior probabilities respectively. The nodes with bootstrap values less than 50% were collapsed. Higher rank names are marked with the vertical black bars.

The concatenated sequences of the exon regions of the 12 EPIC markers totalled 3195 bp, with each marker varying from 207 bp to 324 bp. Bayesian analysis and ML analysis resulted in the same phylogeny (Figure 4). Many well-recognized clades in classic taxonomy were highly supported in the resulting phylogeny, such as Tetraodontiformes, Gobioidei, Cypriniformes and Percomorpha, all marked by the high Bayesian posterior probabilities and bootstrap support (Figure 4). These consistent results in the phylogeny reconstruction suggest that the exon portion of EPIC marker is useful for inferring phylogenetic relationships.

## Discussion

Wiens [25] described a "wish list" for the ideal method in delimiting species. He suggested that such a method "considers both incomplete lineage sorting and gene flow among populations, can integrate data from multiple loci, can determine species limits without having those limits defined *a priori *(i.e., it will allow one to discover unanticipated species from the molecular data), and can allow one to estimate the statistical support for species-level decisions". If we think thoroughly about what kind of data are necessary to achieve such goals, we are always led to multilocus data. For example, many independent loci can sort out the signal of population divergence from gene flow or random lineage sorting. Individual based assignment tests, without the need of defining populations *a priori*, all require multilocus data and the power of those methods is highly dependent on the number of loci available [6,7].

As mitochondrial loci typically are linked in vertebrates, they essentially act as a single locus, which precludes them as the sole data source for multilocus population studies. Single-copy nuclear polymorphic sequences are widely regarded as the marker of choice; but even these markers are not free of obstacles, such as the availability of the markers and technical hurdles in resolving haplotypes [26]. A good nuclear marker for studies at the species-level should be amplifiable across different taxonomic groups and the target sequence should exhibit reasonable variation at the intraspecific level [26]. EPIC markers seem to be a natural fit to this description. The conserved exon portion of EPIC markers improves the versatility of primer amplification across taxa whereas the more variable intron portion provides variation for intraspecific studies. On the other hand, our approach, focusing on single-copy and conserved coding sequence to facilitate universal primers design and homolog identification, is a biased representation of the whole genome. However, the sequence conservation in the exonic part of the gene does not necessarily suggest a slow evolutionary rate in the intronic part of the gene. Nevertheless, one study showed that introns had a low average genetic diversity relative to anonymous loci in birds [3]; another study found a correlation between the evolutionary rate of exons and non-coding regions in *Drosophila melanogaster *[27]. Therefore, the limitation of variation in EPIC marker and the correlation between the rate of its intron and exon portion await more scrutiny from empirical studies.

Nonetheless, two of the obstacles in applying single-copy nuclear polymorphic sequence in population studies can now be overcome due to recent developments in genomics and high throughput sequencing techniques. First, due to the fast accumulation of public accessible genome sequences and EST databases, mining genetic markers *in silico *has never been easier, as shown by this study. Currently, the Ensembl genome browser maintains more than 51 genome assemblages (http://www.ensembl.org, as of Nov 2009), while GenBank holds 63,463,018 EST records (http://www.ncbi.nlm.nih.gov/, as of Nov 2009). Methods based on comparison between two or more genomes or between one genome and the EST sequences have been developed for plants, birds and fishes [16-19,23,28,29]. The worries about the versatility of EPIC markers applying across different taxa [30] and the difficulties in developing such markers [26] should be alleviated now. Due to the limited genomic resources, the early approaches often utilized both the genome and EST sequences available at the time [17,19]. Since EST sequences do not contain the information about introns and are usually incomplete, the EPIC markers developed from EST will likely be of a lower quality, such as multiple copies in the genome and less predictable in the size of the markers, which might require more lab work in screening steps for finding the good markers.

The other technical hurdle for utilizing intron sequences is how to resolve haplotypes. As the markers for studies at the species level, high polymorphism can exist within species and among alleles in EPIC markers. Strictly speaking, the alleles of diploid individuals have to be determined before performing any meaningful species level analysis. There are experimental methods to resolve haplotypes, such as cloning, gel extraction, "allele-dropout-effect" and SSCP [26], all of which demand considerable laboratory effort. Alternatively, haplotypes can be resolved statistically, using the software packages such as PHASE [31]. However, physically separating the two alleles is usually the only choice, if there is length variation between them, so called length variant heterozygote (LVH) [32]. A new solution to separating sequences from two alleles is using the next-generation sequencing. New developments in the next-generation sequencing [33], in theory allow for the two alleles in each individual to be sequenced in parallel simultaneously, avoiding the extensive cloning steps. The current challenge is to design ways of tagging DNA to facilitate sequencing many genes and many individuals in one run [34-36].

We aimed to develop a list of candidate markers for studying closely related species or populations for any group of teleost fish, especially the non-model species. Thus, we designed primers on alignments of all five fish genomes to increase to success rate of amplification in any teleosts. If we were interested in a particular group of teleosts, for example, gasterosteiforms, we could have used *Gasterosteus aculeatus *as the query and use *Oryzias latipes *as the reference to increase the specificity of resulting primers, but those primers might only applicable to that group of fishes. Since our primers were designed on conserved exonic part of the gene, using all five genomes provided good priming sites for designing universal primer, which can be applied to other non-model teleosts. There are only a handful of model organisms for which whole genome sequences are available, so our approach is particularly important for developing markers in non-model organisms. Our results show that several markers amplified distantly related non-model teleosts.

The putative "whole-genome duplication" events at the base of teleosts could have serious impact in ortholog identification. It is especially problematic if differential gene loss happened in different lineages after the genome duplication [23]. Nevertheless, we intended to develop single-copy markers for studying closely related species or populations, so the differential gene loss would have less detrimental effects, i.e., the gene copy is most probably orthologous in closely related species or different individuals of the same species. If one pair of primers resulted in multiple fragments in a particular species possibly due to gene duplication, the marker should be discarded for that species.

In our experiment, we tested 12 candidate markers in nine distantly related teleost fishes to illustrate the broad taxonomic usage of those markers. We found that five to 12 of the markers amplified different species, leaving some missing data. Although this test case served as an illustration for how these markers might work across taxa, it was not a typical study, in which closely related species or populations are examined. In such cases, similar set of loci should amplify equally well in different individuals or species, as shown in our results (Table 3, Figure 3); thus, resulting in less or no missing data.

## Conclusions

We developed an efficient strategy for mining EPIC markers by comparing genome sequences. Applying the bioinformatic tools developed in this study, we found thousands of candidate EPIC markers in teleost fishes. By testing some of the candidate markers, we illustrated the usefulness of these markers in a broad range of teleost taxa. The strategy and the bioinformatic tools we developed are readily adapted for other taxonomic groups.

## Method

### Marker development

**Figure 5:**
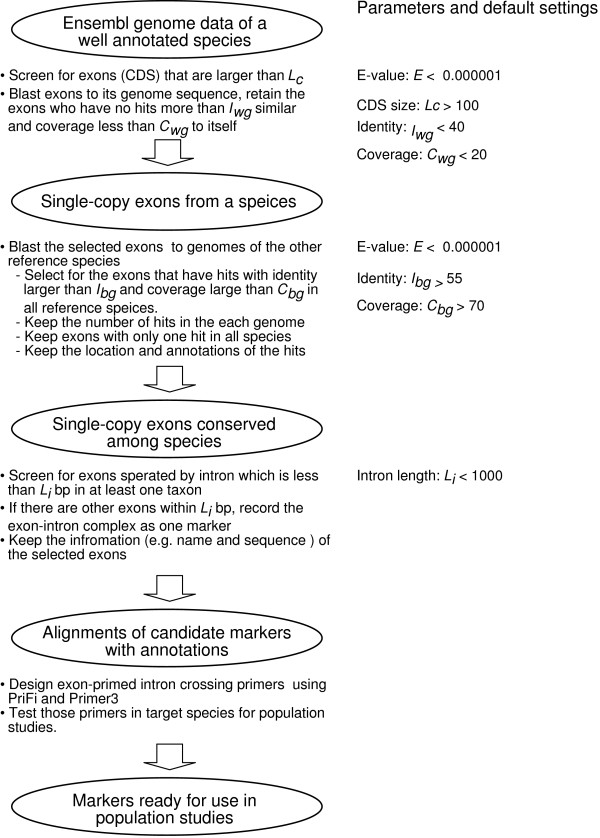
**Pipeline for mining the exon-primed intron-crossing (EPIC) markers**. The input data include the position of coding sequences from a well-annotated query genome, and fasta sequence files of one or more reference genomes. Perl scripts implementing this procedure are available upon request (correspondence to C. Li).

We designed a pipeline to identify short introns (< 1,000 bp in at least one taxon) bounded by single-copy and conserved exons (Figure 5). First, we took a strategy similar to Li *et al*. [23] in spotting the single-copy and conserved exons. Two important modifications were made in this study: (1) the coding sequence (CDS) was used instead of the full exon sequence, because sometimes the 5'- or 3'- untranslated regions (UTR) of the exons are too variable among distantly related taxa, and can obscure the true conserveness between CDS; (2) we lowered the penalty score for mismatch from -3 to -1 in the BLAST [37] alignment step, so the comparison of CDS can be extended over occasionally low-matched regions. Both modifications were essential in achieving better results. After locating the single-copy conserved exons, we screened for exons flanking the intron, which is smaller than 1,000 bp in at least one of the compared genomes, in order to facilitate the subsequent PCR and sequencing steps (Figure 5). If multiple EPIC markers are present within the range of 1,000 bp, we report the whole region as one marker. After being identified as candidate markers, the fasta sequences of the markers were retrieved from each genome. All of the above steps were automated using Perl scripts.

The exon portion of each sequence was aligned using ClustalW [38] implemented in MEGA4 [39]. PriFi [40] was used to find the optimal regions for designing primers on the aligned sequences. Primers were designed by eye and checked with Primer3 [41]. Whenever possible, the 3' end of the primers was designed on the 1^st ^or 2^nd ^codon position and the number of mismatches in the last five nucleotides of 3' end of the primers also was minimized, in order to increase annealing accuracy of the primers.

### In vitro marker validation

Twelve randomly selected EPIC markers were tested in nine teleost fishes (Table 1 and 2). The species related to the current research interests of the authors were used, but they also were chosen to cover a broad phylogenetic range. *Danio rerio *was used as the positive control. In addition, two other cypriniforms (*Hypophthalmichthys molitrix *and *H. nobilis*), one clupeiforms (*Thryssa hamiltonii*) and five perciforms (*Larimichthys crocea*, *Sinperca chuatsi*, *Odontobutis potamophila*, *Micropercops swinhonis *and *Rhynogobius giurinus*) were picked. As deliberately designed, we had a taxon (*T. hamiltonii*) diverged from the others at about 307 million years ago [42], several families, two closely related genera (*Odontobutis *and *Micropercops*) and two congeneric species (*H. molitrix *and *H. nobilis*), so the selected taxa covered a broad range of genetic distance.

DNA samples were extracted from muscle tissues or fin clips using a standard phenol-chloroform methods [43]. All PCR were performed in a total volume of 20 μl, including 0.1 μl TaKaRa Taq™ (Takara, Shanghai, China), 2.0 μl 10 × PCR buffer (+ MgCl_2_), 1.6 μl dNTP Mixture (2.5 mM each dNTP), 0.64 μl forward and reverse primers respectively, 0.8 μl DNA template and 14.22 μl distilled water. The PCR reactions were carried out in an Eppendorf Mastercycler with silver block (Eppendorf China Ltd, Shanghai, China). The PCR program consisted of a 95°C initial heating for 30 sec, 15 cycles of 98°C for 10 sec, 60°C for 30 sec and 72°C for 45 sec, 15 cycles of 98°C for 10 sec, 58°C for 30 sec and 72°C for 45 sec, followed by a final extension at 72°C for 5 min. The PCR products were visualized on agarose gels. The amplified products were sequenced by Shanghai Sangon Biological Engineering Technology & Services Co, Ltd. (Shanghai, China).

### Data analysis

The sequences determined in this study and the sequences of the model species retrieved from the Ensembl database were aligned to each other via ClustalW [38]. Because of the large genetic distance among most tested species, the intron sequences were not alignable except for the congeneric species (*H. molitrix *and *H. nobilis*) and two closely related genera (*O. potamophila *and *M. swinhonis*). Therefore, the alignment was made only on the exon parts of EPIC sequences and the size of introns was recorded. The exon sequences were translated into amino acid to be aligned using ClustalW [38] implemented in MEGA4 [39]; then, the aligned sequences were translated back into nucleotides.

Pairwise *p*-distances [44] were estimated for all taxa based on the concatenated exon sequences of the 12 EPIC markers using MEGA4 [39]. The Pearson correlation coefficient between the difference in intron size and the genetic distance of every taxon-pair was estimated using the SAS program (SAS Institute Inc., Cary, NC, USA). Similarly, the correlation coefficient between the difference in amplifiability for the same suite of markers in two taxa and the genetic distance between them was also calculated. The difference in amplifiability for taxon *i *and taxon *j *was defined as:

in which, *DA_ij _*is the difference of amplifiability between taxon *i *and taxon *j*;  is the number of markers amplified in both taxon *i *and taxon *j*, while  is the number of markers amplified in any of the two taxa. The value of *DA_ij _*could range from 0 (no shared markers) to 1 (all markers are shared). One important practical question we might have is that if we know certain EPIC markers worked for one species, can we apply the same markers on other species? Or how close two species should be related to ensure the same markers work? We calculated the correlation between the amplifiability and the genetic distance among species, and the correlation between the intron size and the genetic distance in order to address this question.

Finally, we tested the utility of the exon parts of the 12 EPIC markers in reconstructing the species phylogeny. The exon sequences from 13 species (9 determined in this study plus 4 retrieved from Ensembl) were concatenated. The concatenated sequences were partitioned by codon positions, because the exon sequences collected for each gene were short (≈ 200 - 300 bp) in this study and not enough sites could be used if we partition them by both codon and gene. It also has been shown that most heterogeneity often could be captured through partitioning by codon position [45]. The optimal model for each data partition was selected by using the "*propose model*" analysis in TreeFinder [46]. Partitioned Bayesian analyses were carried out using MrBayes [47]. The closest models to the TreeFinder selected models were applied in Bayesian analysis. Two independent runs, with 8 Markov Chain Monte Carlo (MCMC) per run were performed for Bayesian analysis. The heating parameter was set as "*temp = 0.1*" to improve the mixing of the MCMC. The runs were terminated after 10 million generations with a sampling frequency of 1 in 1000 (10,000 trees save for each run). After discarding the *burnin *samples (1000 trees from each run), 50% majority rule tree was calculated using *sumt*. Maximum likelihood phylogeny was searched using TreeFinder using the best models for each data partition. Bootstrap analysis with 1000 replications was performed to assess the statistical support for each node of the ML tree. The resulting phylogeny was graphed using Dendroscope [48].

## Authors' contributions

CL developed the bioinformatic pipeline, wrote the scripts, analyzed the data and wrote the paper. LM tested the EPIC markers and collected sequences. J-JMR helped with the Perl scripts and writing. All authors have read and approved the final manuscript.

## Supplementary Material

Additional file 1 **The gene description, chromosomal location and start position of EPIC markers identified in this study**. The additional file 1 lists 210 EPIC markers found in this study, whose exon portion has average identity larger than 85%. The markers are named using the last five numbers of the Ensembl gene name plus a number that distinguishing markers found in the same gene. The location information shown is from *D. rerio*. The first 12 markers were tested in nine teleost fishes. Click here for file
