# Supplementary material for: Exon-primed intron-crossing (EPIC) markers for non-model teleost fishes
Source: BMC Evol Biol. 2010 Mar 31;10:90. doi: 10.1186/1471-2148-10-90 (PMC2853542; doi:10.1186/1471-2148-10-90)
Supplement: Additional file 1 — The gene description, chromosomal location and start position of EPIC markers identified in this study. The additional file 1 lists 210 EPIC markers found in this study, whose exon portion has average identity larger than 85%. The markers are named using the last five numbers of the Ensembl gene name plus a number that distinguishing markers found in the same gene. The location information shown is from D. rerio. The first 12 markers were tested in nine teleost fishes. [file 1471-2148-10-90-S1.DOC]

### Additional file 1

| Locus | | Chromosome | Marker start (bp) | | Gene description | |
| --- | --- | --- | --- | --- | --- | --- |
| 59107E2 | | 4 | 572793 | | UPF0027 protein C22orf28 homolog. [Source:Uniprot/SWISSPROT;Acc:Q6NZS4] | |
| 55378E1 | | Zv7_NA122 | 96106 | | Peroxisome proliferator activated receptor gamma coactivator 1 alpha (Fragment). [Source:Uniprot/SPTREMBL;Acc:Q52MY8] | |
| 55305E1 | | 13 | 26342049 | | ret proto-oncogene [Source:RefSeq peptide;Acc:NP_858048] | |
| 40245E5 | | 1 | 2590224 | | hypothetical protein LOC569455 [Source:RefSeq peptide;Acc:NP_001139076] | |
| 36298E1 | | 7 | 18108484 | | hypothetical protein LOC415169 [Source:RefSeq_peptide;Acc:NP_001002079] | |
| 25073E1 | | 4 | 1723855 | | 60S ribosomal protein L18a [Source:UniProtKB/Swiss- Prot;Acc:Q7ZWJ4] | |
| 19231E4 | | 21 | 10663833 | | spectrin alpha 2 [Source:RefSeq peptide;Acc:NP_001091958] | |
| 14867E1 | | 9 | 33724200 | | 60S ribosomal protein L8 [Source:UniProtKB/Swiss- Prot;Acc:Q6P0V6] | |
| 08680E3 | | 19 | 45181216 | | karyopherin (importin) beta 1 [Source:RefSeq_peptide;Acc:NP_001032791] | |
| 08680E2 | | 19 | 45180803 | | karyopherin (importin) beta 1 [Source:RefSeq_peptide;Acc:NP_001032791] | |
| 04174E20 | | 25 | 13957143 | | CCR4-NOT transcription complex subunit 1 (CCR4- associated factor 1) [Source:UniProtKB/Swiss- Prot;Acc:A1A5H6] | |
| 01777E4 | | 10 | 2555539 | | nucleoporin 155 [Source:RefSeq peptide;Acc:NP_956450] | |
| 68830E2 | | 4 | 66911816 | | hypothetical protein LOC100003412 [Source:RefSeq peptide;Acc:NP_001121760] | |
| 68830E1 | | Zv7_NA164 | 5026 | | Uncharacterized protein C22orf9. [Source:Uniprot/SWISSPROT;Acc:Q6ICG6] | |
| 42642E1 | | 20 | 30650894 | | Pre-mRNA-splicing regulator WTAP (Wilms tumor 1- associating protein)(WT1-associated protein)(Female- lethal(2)D homolog) [Source:UniProtKB/Swiss- Prot;Acc:Q7SXL7] | |
| 41920E6 | | 8 | 33499582 | | hypothetical protein LOC436693 [Source:RefSeq_peptide;Acc:NP_001002420] | |
| 35692E1 | | 1 | 24745106 | | 40S ribosomal protein S3a [Source:UniProtKB/Swiss- Prot;Acc:Q6PBY1] | |
| 35564E5 | | 5 | 22556317 | | DiGeorge syndrome critical region gene 8 [Source:RefSeq peptide;Acc:NP_001116221] | |
| 25073E2 | | 4 | 165576 | | 60S ribosomal protein L18a. [Source:Uniprot/SWISSPROT;Acc:Q7ZWJ4] | |
| 19000E1 | | 22 | 32398436 | | chondroitin sulfate proteoglycan 6 [Source:RefSeq peptide;Acc:NP_999854] | |
| 18049E2 | | 7 | 6279648 | | hypothetical protein LOC570994 [Source:RefSeq peptide;Acc:NP_001098747] | |
| 16496E1 | | 24 | 20125999 | | cyclin-dependent kinase 8 [Source:RefSeq_peptide;Acc:NP_919389] | |
| 14867E4 | | 9 | 33729639 | | 60S ribosomal protein L8 [Source:UniProtKB/Swiss- Prot;Acc:Q6P0V6] | |
| 14690E1 | | 7 | 50255573 | |  | |
| 12848E4 | | 8 | 26817662 | | ariadne homolog 2 [Source:RefSeq peptide;Acc:NP_998308] | |
| 07216E4 | | 23 | 39851777 | |  | |
| 01835E6 | | 9 | 32001495 | |  | |
| 53047E2 | | 6 | 53535701 | |  | |
| 19230E4 | | 5 | 31613756 | | ribosomal protein L7a [Source:RefSeq peptide;Acc:NP_956341] | |
| 26908E1 | | 10 | 3714513 | | transmembrane emp24 domain trafficking protein 2 [Source:RefSeq peptide;Acc:NP_955842] | |
| 79949E3 | | 7 | 2826914 | | suppressor of Ty 16 homolog [Source:RefSeq peptide;Acc:NP_001091053] | |
| 19000E4 | | 22 | 32406534 | | chondroitin sulfate proteoglycan 6 [Source:RefSeq peptide;Acc:NP_999854] | |
| 06729E1 | | 13 | 43747195 | | Eukaryotic translation initiation factor 3 subunit L (eIF3l)(Eukaryotic translation initiation factor 3 subunit E-interacting protein)(Eukaryotic translation initiation factor 3 subunit 6 | |
| 06729E2 | | 13 | 43748652 | | Eukaryotic translation initiation factor 3 subunit L (eIF3l)(Eukaryotic translation initiation factor 3 subunit E-interacting protein)(Eukaryotic translation initiation factor 3 subunit 6 | |
| 14867E2 | | 9 | 33726004 | | 60S ribosomal protein L8 [Source:UniProtKB/Swiss- Prot;Acc:Q6P0V6] | |
| 73975E13 | | 13 | 6769719 | |  | |
| 12592E1 | | 14 | 52668349 | | RNA polymerase II subunit B Fragment [Source:UniProtKB/TrEMBL;Acc:Q6DRH1] | |
| 26180E26 | | 15 | 28787547 | | pre-mRNA processing factor 8 [Source:RefSeq peptide;Acc:NP_957270] | |
| 35692E3 | | 1 | 24744510 | | 40S ribosomal protein S3a [Source:UniProtKB/Swiss- Prot;Acc:Q6PBY1] | |
| 40245E10 | | 1 | 2591037 | | hypothetical protein LOC569455 [Source:RefSeq peptide;Acc:NP_001139076] | |
| 22974E5 | | 8 | 6803939 | |  | |
| 42905E2 | | 8 | 24507401 | | 60S ribosomal protein L10a [Source:UniProtKB/Swiss- Prot;Acc:Q6PC69] | |
| 34823E3 | | 4 | 15799546 | | Coatomer subunit gamma-2 (Gamma-2-coat protein)(Gamma-2-COP) [Source:UniProtKB/Swiss- Prot;Acc:Q9PUE4] | |
| 34823E2 | | 4 | 15798554 | | Coatomer subunit gamma-2 (Gamma-2-coat protein)(Gamma-2-COP) [Source:UniProtKB/Swiss- Prot;Acc:Q9PUE4] | |
| 67913E1 | | Zv8_NA11417 | 46821 | | hypothetical protein LOC768124 [Source:RefSeq peptide;Acc:NP_001070738] | |
| 59622E2 | | 3 | 39816141 | | hypothetical protein LOC562437 [Source:RefSeq peptide;Acc:NP_001076299] | |
| 40548E1 | | 1 | 816430 | | Polycomb protein eed [Source:UniProtKB/Swiss- Prot;Acc:Q566T0] | |
| 54213E1 | | 17 | 26341846 | | HECT domain containing 1 [Source:RefSeq peptide;Acc:NP_001002504] | |
| 61397E13 | | 18 | 41368701 | | Novel protein similar to vertebrate thyroid hormone receptor interactor 12 (TRIP12) Fragment [Source:UniProtKB/TrEMBL;Acc:A3KPN5] | |
| 62933E1 | | 18 | 13262399 | | Novel protein similar to human c-Maf-inducing protein (CMIP) Fragment [Source:UniProtKB/TrEMBL;Acc:Q1LWW3] | |
| 30756E5 | | 3 | 53499048 | | DNA (cytosine-5-)-methyltransferase 1 [Source:RefSeq peptide;Acc:NP_571264] | |
| 03449E3 | | 13 | 4704616 | | phosphodiesterase 10A [Source:RefSeq peptide;Acc:NP_957396] | |
| 57556E1 | | 21 | 20088037 | | hypothetical protein LOC336641 [Source:RefSeq peptide;Acc:NP_997925] | |
| 61719E2 | | 25 | 29958428 | |  | |
| 01777E8 | | 10 | 2556787 | | nucleoporin 155 [Source:RefSeq peptide;Acc:NP_956450] | |
| 79949E1 | | 7 | 2818993 | | suppressor of Ty 16 homolog [Source:RefSeq peptide;Acc:NP_001091053] | |
| 45228E1 | | 24 | 38236326 | | STIP1 homology and U-box containing protein 1 [Source:RefSeq peptide;Acc:NP_955968] | |
| 78653E1 | | Zv8_NA3195 | 937 | | hypothetical protein LOC404612 [Source:RefSeq peptide;Acc:NP_996963] | |
| 40853E1 | | 22 | 32134276 | | hypothetical protein LOC555476 [Source:RefSeq peptide;Acc:NP_001038251] | |
| 04174E1 | | 25 | 13939478 | | CCR4-NOT transcription complex subunit 1 (CCR4- associated factor 1) [Source:UniProtKB/Swiss- Prot;Acc:A1A5H6] | |
| 14690E1 | | 7 | 52467778 | |  | |
| 63147E4 | | 25 | 6548827 | |  | |
| 27825E1 | | 24 | 20762581 | | N-acetyltransferase NAT13 (EC 2.3.1.-) [Source:UniProtKB/Swiss-Prot;Acc:Q6DBY2] | |
| 04174E19 | | 25 | 13956914 | | CCR4-NOT transcription complex subunit 1 (CCR4- associated factor 1) [Source:UniProtKB/Swiss- Prot;Acc:A1A5H6] | |
| 75097E10 | | 23 | 28017082 | |  | |
| 26180E29 | | 15 | 28788394 | | pre-mRNA processing factor 8 [Source:RefSeq peptide;Acc:NP_957270] | |
| 54829E1 | | 7 | 24660494 | |  | |
| 42658E1 | | 15 | 19207386 | |  | |
| 26180E7 | | 15 | 28780496 | | pre-mRNA processing factor 8 [Source:RefSeq peptide;Acc:NP_957270] | |
| 18049E1 | | 7 | 6267171 | | hypothetical protein LOC570994 [Source:RefSeq peptide;Acc:NP_001098747] | |
| 43509E1 | | 19 | 43139828 | | ribosomal protein L11 [Source:RefSeq peptide;Acc:NP_001002139] | |
| 79771E1 | | 7 | 76298346 | |  | |
| 26180E8 | | 15 | 28780823 | | pre-mRNA processing factor 8 [Source:RefSeq peptide;Acc:NP_957270] | |
| 06729E3 | | 13 | 43750697 | | Eukaryotic translation initiation factor 3 subunit L (eIF3l)(Eukaryotic translation initiation factor 3 subunit E-interacting protein)(Eukaryotic translation initiation factor 3 subunit 6 | |
| 21557E3 | 12 | | | 168169 | | WD repeat domain phosphoinositide-interacting protein 3 (WIPI-3)(WD repeat-containing protein 45- like)(WDR45-like protein) [Source:UniProtKB/Swiss- Prot;Acc:Q7ZUW6] |
| 25220E2 | 10 | | | 17303471 | | signal peptide peptidase 3 [Source:RefSeq peptide;Acc:NP_001015068] |
| 75624E1 | 8 | | | 15670270 | | Novel protein Fragment [Source:UniProtKB/TrEMBL;Acc:B0UYU2] |
| 62330E1 | 13 | | | 12987623 | | hypothetical protein LOC564367 [Source:RefSeq peptide;Acc:NP_001139056] |
| 62915E7 | 18 | | | 15366354 | | DNA-directed RNA polymerase (EC 2.7.7.6) [Source:UniProtKB/TrEMBL;Acc:A2BG99] |
| 13965E6 | 24 | | | 2144884 | |  |
| 25576E5 | 4 | | | 11321414 | |  |
| 25212E3 | 23 | | | 43042559 | | Integrator complex subunit 11 (Int11)(EC 3.1.27.- )(Cleavage and polyadenylation-specific factor 3-like protein)(CPSF3-like protein) [Source:UniProtKB/Swiss- Prot;Acc:Q503E1] |
| 77536E8 | 8 | | | 31770063 | | similar to Activating signal cointegrator 1 complex subunit 3-like 1 [Source:RefSeq peptide;Acc:NP_001116729] |
| 19000E7 | 22 | | | 32416941 | | chondroitin sulfate proteoglycan 6 [Source:RefSeq peptide;Acc:NP_999854] |
| 34823E4 | 4 | | | 15800353 | | Coatomer subunit gamma-2 (Gamma-2-coat protein)(Gamma-2-COP) [Source:UniProtKB/Swiss- Prot;Acc:Q9PUE4] |
| 63100E1 | 11 | | | 8859285 | | hypothetical protein LOC799058 [Source:RefSeq peptide;Acc:NP_001077042] |
| 35692E2 | 1 | | | 24743302 | | 40S ribosomal protein S3a [Source:UniProtKB/Swiss- Prot;Acc:Q6PBY1] |
| 39641E1 | 14 | | | 52584945 | | ribosomal protein L26 [Source:RefSeq peptide;Acc:NP_998278] |
| 18190E2 | 1 | | | 52023323 | | Arsenical pump-driving ATPase (EC 3.6.3.16)(Arsenite-translocating ATPase)(Arsenical resistance ATPase)(Arsenite-transporting ATPase) [Source:UniProtKB/Swiss-Prot;Acc:Q6IQE5] |
| 62915E6 | 18 | | | 15365586 | | DNA-directed RNA polymerase (EC 2.7.7.6) [Source:UniProtKB/TrEMBL;Acc:A2BG99] |
| 53196E2 | 8 | | | 46695738 | | FK506 binding protein 12-rapamycin associated protein 1 [Source:UniProtKB/TrEMBL;Acc:B0UX67] |
| 29157E7 | 20 | | | 2464723 | | Mediator of RNA polymerase II transcription subunit 23 (Mediator complex subunit 23)(Cofactor required for Sp1 transcriptional activation subunit 3)(CRSP complex subunit 3) [Source:UniProtKB/Sw |
| 12592E7 | 14 | | | 52675636 | | RNA polymerase II subunit B Fragment [Source:UniProtKB/TrEMBL;Acc:Q6DRH1] |
| 15905E2 | 5 | | | 9936646 | | leucine-zipper-like transcription regulator 1 [Source:RefSeq peptide;Acc:NP_001074074] |
| 03167E5 | 12 | | | 17561312 | | Protein LST8 homolog [Source:UniProtKB/Swiss- Prot;Acc:Q803V5] |
| 07901E2 | Zv8_scaffold2957 | | | 34356 | | hypothetical protein LOC393920 [Source:RefSeq peptide;Acc:NP_957240] |
| 03449E1 | 13 | | | 4679439 | | phosphodiesterase 10A [Source:RefSeq peptide;Acc:NP_957396] |
| 59070E1 | 24 | | | 20120424 | | Wu:fb02f03 protein Fragment [Source:UniProtKB/TrEMBL;Acc:Q4V9I4] |
| 12592E6 | 14 | | | 52673746 | | RNA polymerase II subunit B Fragment [Source:UniProtKB/TrEMBL;Acc:Q6DRH1] |
| 62558E2 | 18 | | | 18081016 | | hypothetical protein LOC565154 [Source:RefSeq peptide;Acc:NP_001038537] |
| 19507E4 | 3 | | | 23101492 | | MCM5 minichromosome maintenance deficient 5 [Source:RefSeq peptide;Acc:NP_848523] |
| 26180E6 | 15 | | | 28780063 | | pre-mRNA processing factor 8 [Source:RefSeq peptide;Acc:NP_957270] |
| 29114E5 | 16 | | | 30901722 | | U4/U6 small nuclear ribonucleoprotein Prp31 (Pre- mRNA-processing factor 31) [Source:UniProtKB/Swiss- Prot;Acc:Q7SXM7] |
| 70849E1 | 11 | | | 5621547 | | ribosomal protein S15 [Source:RefSeq peptide;Acc:NP_001001819] |
| 31915E1 | 13 | | | 29461726 | | Hypoxia-inducible factor 1-alpha inhibitor (EC 1.14.11.16)(Hypoxia-inducible factor asparagine hydroxylase) [Source:UniProtKB/Swiss-Prot;Acc:P59723] |
| 19778E2 | 1 | | | 43563257 | | ribosomal protein S6 [Source:RefSeq peptide;Acc:NP_001003728] |
| 18904E5 | 18 | | | 44609952 | |  |
| 26180E25 | 15 | | | 28786487 | | pre-mRNA processing factor 8 [Source:RefSeq peptide;Acc:NP_957270] |
| 62330E23 | 13 | | | 13131031 | | hypothetical protein LOC564367 [Source:RefSeq peptide;Acc:NP_001139056] |
| 12592E3 | 14 | | | 52669284 | | RNA polymerase II subunit B Fragment [Source:UniProtKB/TrEMBL;Acc:Q6DRH1] |
| 42724E3 | 15 | | | 20548590 | | Transcription elongation factor SPT5 (DRB sensitivity-inducing factor large subunit)(DSIF large subunit)(Protein foggy) [Source:UniProtKB/Swiss- Prot;Acc:Q9DDT5] |
| 29157E8 | 20 | | | 2469379 | | Mediator of RNA polymerase II transcription subunit 23 (Mediator complex subunit 23)(Cofactor required for Sp1 transcriptional activation subunit 3)(CRSP complex subunit 3) [Source:UniProtKB/Sw |
| 13965E1 | 24 | | | 2132072 | |  |
| 01835E4 | 9 | | | 31999045 | |  |
| 63100E2 | 11 | | | 8867738 | | hypothetical protein LOC799058 [Source:RefSeq peptide;Acc:NP_001077042] |
| 26180E9 | 15 | | | 28781109 | | pre-mRNA processing factor 8 [Source:RefSeq peptide;Acc:NP_957270] |
| 03963E1 | 15 | | | 14263514 | | tnf receptor-associated factor 4a [Source:RefSeq peptide;Acc:NP_991325] |
| 45680E6 | 4 | | | 13202671 | | transportin 3 [Source:RefSeq peptide;Acc:NP_957381] |
| 23113E2 | 8 | | | 47551837 | | yippee-like 5 [Source:RefSeq peptide;Acc:NP_956771] |
| 18192E20 | 16 | | | 49419560 | | Ubr5 protein Fragment [Source:UniProtKB/TrEMBL;Acc:Q642I4] |
| 01220E19 | 9 | | | 15818310 | | MYC binding protein 2 [Source:RefSeq peptide;Acc:NP_001012247] |
| 15905E1 | 5 | | | 9936083 | | leucine-zipper-like transcription regulator 1 [Source:RefSeq peptide;Acc:NP_001074074] |
| 68067E2 | 5 | | | 29203252 | | Novel protein Fragment [Source:UniProtKB/TrEMBL;Acc:A2CES2] |
| 09549E45 | 23 | | | 20847086 | |  |
| 56138E2 | 9 | | | 32569267 | |  |
| 36190E2 | 19 | | | 19453664 | | hypothetical protein LOC449780 [Source:RefSeq peptide;Acc:NP_001005953] |
| 07092E3 | 3 | | | 50862755 | | hypothetical protein LOC555321 [Source:RefSeq peptide;Acc:NP_001038248] |
| 41619E1 | 14 | | | 6450827 | | Guanine nucleotide-binding protein subunit beta-2- like 1 (Receptor of activated protein kinase C)(RACK) [Source:UniProtKB/Swiss-Prot;Acc:O42248] |
| 60442E1 | 11 | | | 13126358 | |  |
| 03151E1 | 21 | | | 46070103 | | S-phase kinase-associated protein 1 [Source:RefSeq peptide;Acc:NP_957037] |
| 77656E1 | 13 | | | 2498017 | | Novel protein similar to vertebrate brefeldin A- inhibited guanine nucleotide-exchange protein 3 (KIAA1244) Fragment [Source:UniProtKB/TrEMBL;Acc:B8JHV8] |
| 53196E13 | 8 | | | 46552852 | | FK506 binding protein 12-rapamycin associated protein 1 [Source:UniProtKB/TrEMBL;Acc:B0UX67] |
| 79949E4 | 7 | | | 2829060 | | suppressor of Ty 16 homolog [Source:RefSeq peptide;Acc:NP_001091053] |
| 18190E1 | 1 | | | 52016309 | | Arsenical pump-driving ATPase (EC 3.6.3.16)(Arsenite-translocating ATPase)(Arsenical resistance ATPase)(Arsenite-transporting ATPase) [Source:UniProtKB/Swiss-Prot;Acc:Q6IQE5] |
| 17190E6 | 16 | | | 41367390 | | Strumpellin [Source:UniProtKB/Swiss- Prot;Acc:Q7ZVM1] |
| 42905E1 | 8 | | | 24507152 | | 60S ribosomal protein L10a [Source:UniProtKB/Swiss- Prot;Acc:Q6PC69] |
| 36875E1 | 23 | | | 30981455 | | ribosomal protein S12 [Source:RefSeq peptide;Acc:NP_956340] |
| 60354E1 | 2 | | | 35278486 | | hypothetical protein LOC563729 [Source:RefSeq peptide;Acc:NP_001122188] |
| 53196E7 | 8 | | | 46731294 | | FK506 binding protein 12-rapamycin associated protein 1 [Source:UniProtKB/TrEMBL;Acc:B0UX67] |
| 08279E2 | 1 | | | 43377843 | |  |
| 10516E1 | 24 | | | 21182120 | | ribosomal protein L21 [Source:RefSeq peptide;Acc:NP_001002155] |
| 19000E6 | 22 | | | 32409684 | | chondroitin sulfate proteoglycan 6 [Source:RefSeq peptide;Acc:NP_999854] |
| 55581E3 | Zv8_scaffold3086 | | | 56173 | | E3 ubiquitin-protein ligase MIB1 (EC 6.3.2.-)(Mind bomb homolog 1)(DAPK-interacting protein 1)(DIP-1)(Zinc finger ZZ type with ankyrin repeat domain protein 2) [Source:UniProtKB |
| 19000E10 | 22 | | | 32422678 | | chondroitin sulfate proteoglycan 6 [Source:RefSeq peptide;Acc:NP_999854] |
| 35956E1 | 19 | | | 30520054 | | hypothetical protein LOC553664 [Source:RefSeq peptide;Acc:NP_001018473] |
| 74003E1 | | 17 | 17196163 | |  | |
| 14500E5 | | 23 | 5683954 | | PRP6 pre-mRNA processing factor 6 [Source:RefSeq peptide;Acc:NP_997820] | |
| 74658E4 | | Zv8_NA2362 | 9262 | | Regulator of nonsense transcripts 2 (Nonsense mRNA reducing factor 2)(Up-frameshift suppressor 2 homolog)(hUpf2) [Source:UniProtKB/Swiss-Prot;Acc:Q9HAU5] | |
| 34916E2 | | 9 | 3696349 | | histone acetyltransferase 1 [Source:RefSeq peptide;Acc:NP_001004572] | |
| 36629E1 | | 12 | 31808556 | | ribosomal protein S14 [Source:RefSeq peptide;Acc:NP_956320] | |
| 19962E3 | | 16 | 31968584 | | Dopey1 protein Fragment [Source:UniProtKB/TrEMBL;Acc:Q5BLI9] | |
| 04280E1 | | 19 | 12222110 | | sec13-like protein [Source:RefSeq peptide;Acc:NP_956217] | |
| 15905E9 | | 5 | 9948090 | | leucine-zipper-like transcription regulator 1 [Source:RefSeq peptide;Acc:NP_001074074] | |
| 40245E8 | | 1 | 2588470 | | hypothetical protein LOC569455 [Source:RefSeq peptide;Acc:NP_001139076] | |
| 43359E4 | | 14 | 50176773 | | required for meiotic nuclear division 5 homolog B [Source:RefSeq peptide;Acc:NP_957068] | |
| 36875E2 | | 23 | 30981766 | | ribosomal protein S12 [Source:RefSeq peptide;Acc:NP_956340] | |
| 53196E9 | | 8 | 46740913 | | FK506 binding protein 12-rapamycin associated protein 1 [Source:UniProtKB/TrEMBL;Acc:B0UX67] | |
| 27187E2 | | 3 | 29940468 | | MYST histone acetyltransferase 1 [Source:RefSeq peptide;Acc:NP_997907] | |
| 38801E1 | | 2 | 32100810 | | kelch-like 20 [Source:RefSeq peptide;Acc:NP_998166] | |
| 38882E10 | | 15 | 1638832 | | structural maintenance of chromosomes 4 [Source:RefSeq peptide;Acc:NP_775360] | |
| 13965E5 | | 24 | 2142833 | |  | |
| 04174E2 | | 25 | 13940130 | | CCR4-NOT transcription complex subunit 1 (CCR4- associated factor 1) [Source:UniProtKB/Swiss- Prot;Acc:A1A5H6] | |
| 53439E6 | | 21 | 47130619 | | Probable ATP-dependent RNA helicase DDX46 (EC 3.6.1.-)(DEAD box protein 46) [Source:UniProtKB/Swiss- Prot;Acc:Q4TVV3] | |
| 58105E1 | | 5 | 20540699 | | 60S ribosomal protein L36a [Source:UniProtKB/Swiss- Prot;Acc:P61485] | |
| 24317E6 | | 3 | 5671264 | | TNF receptor-associated protein 1 [Source:RefSeq peptide;Acc:NP_001107097] | |
| 45228E2 | | 24 | 38238085 | | STIP1 homology and U-box containing protein 1 [Source:RefSeq peptide;Acc:NP_955968] | |
| 61719E3 | | 25 | 29961560 | |  | |
| 79848E3 | | 18 | 34324360 | | guanine monophosphate synthetase [Source:RefSeq peptide;Acc:NP_956881] | |
| 03570E5 | | 4 | 9491239 | |  | |
| 63573E1 | | 24 | 9646911 | |  | |
| 57430E1 | | 15 | 45225766 | | Putative uncharacterized protein Fragment [Source:UniProtKB/TrEMBL;Acc:Q498Q6] | |
| 53196E8 | | 8 | 46732738 | | FK506 binding protein 12-rapamycin associated protein 1 [Source:UniProtKB/TrEMBL;Acc:B0UX67] | |
| 12848E5 | | 8 | 26819028 | | ariadne homolog 2 [Source:RefSeq peptide;Acc:NP_998308] | |
| 16443E1 | | 12 | 4262920 | | Eukaryotic translation initiation factor 3 subunit C (eIF3c)(Eukaryotic translation initiation factor 3 subunit 8) [Source:UniProtKB/Swiss-Prot;Acc:Q6PFQ2] | |
| 14867E3 | | 9 | 33726310 | | 60S ribosomal protein L8 [Source:UniProtKB/Swiss- Prot;Acc:Q6P0V6] | |
| 36232E1 | | 18 | 201467 | | Transient receptor potential cation channel subfamily M member 7 (EC 2.7.11.1)(Long transient receptor potential channel 7)(LTrpC7)(Channel-kinase 1) [Source:UniProtKB/Swiss-Prot;Acc:Q96QT4] | |
| 19231E1 | | 21 | 10643817 | | spectrin alpha 2 [Source:RefSeq peptide;Acc:NP_001091958] | |
| 76473E2 | | 10 | 30912616 | |  | |
| 26180E12 | | 15 | 28781966 | | pre-mRNA processing factor 8 [Source:RefSeq peptide;Acc:NP_957270] | |
| 04174E16 | | 25 | 13951884 | | CCR4-NOT transcription complex subunit 1 (CCR4- associated factor 1) [Source:UniProtKB/Swiss- Prot;Acc:A1A5H6] | |
| 26180E13 | | 15 | 28782211 | | pre-mRNA processing factor 8 [Source:RefSeq peptide;Acc:NP_957270] | |
| 19231E5 | | 21 | 10664945 | | spectrin alpha 2 [Source:RefSeq peptide;Acc:NP_001091958] | |
| 21945E1 | | 23 | 13836939 | | DEAD (Asp-Glu-Ala-Asp) box polypeptide 23 [Source:RefSeq peptide;Acc:NP_956176] | |
| 18909E1 | | 25 | 38744719 | | hypothetical protein LOC336726 [Source:RefSeq peptide;Acc:NP_956324] | |
| 42724E2 | | 15 | 20546846 | | Transcription elongation factor SPT5 (DRB sensitivity-inducing factor large subunit)(DSIF large subunit)(Protein foggy) [Source:UniProtKB/Swiss- Prot;Acc:Q9DDT5] | |
| 77331E6 | | 13 | 49203592 | |  | |
| 04174E15 | | 25 | 13951339 | | CCR4-NOT transcription complex subunit 1 (CCR4- associated factor 1) [Source:UniProtKB/Swiss- Prot;Acc:A1A5H6] | |
| 26180E5 | | 15 | 28779744 | | pre-mRNA processing factor 8 [Source:RefSeq peptide;Acc:NP_957270] | |
| 37838E3 | | 3 | 42866198 | | RING finger protein unkempt-like [Source:UniProtKB/Swiss-Prot;Acc:Q9H9P5] | |
| 12592E2 | | 14 | 52668875 | | RNA polymerase II subunit B Fragment [Source:UniProtKB/TrEMBL;Acc:Q6DRH1] | |
| 71087E1 | | 25 | 2463429 | | UDP-xylose and UDP-N-acetylglucosamine transporter (Solute carrier family 35 member B4) [Source:UniProtKB/Swiss-Prot;Acc:Q7ZW46] | |
| 19231E3 | | 21 | 10652151 | | spectrin alpha 2 [Source:RefSeq peptide;Acc:NP_001091958] | |
| 08224E10 | | 7 | 43353509 | | Novel protein simlar to human and mouse vacuolar protein sorting 35 (Yeast) (VPS35)Zgc:136268 protein ; [Source:UniProtKB/TrEMBL;Acc:Q6ZM34] | |
| 62330E19 | | 13 | 13123708 | | hypothetical protein LOC564367 [Source:RefSeq peptide;Acc:NP_001139056] | |
| 77536E7 | | 8 | 31769861 | | similar to Activating signal cointegrator 1 complex subunit 3-like 1 [Source:RefSeq peptide;Acc:NP_001116729] | |
| 06729E5 | | 13 | 43753034 | | Eukaryotic translation initiation factor 3 subunit L (eIF3l)(Eukaryotic translation initiation factor 3 subunit E-interacting protein)(Eukaryotic translation initiation factor 3 subunit 6 | |
| 26180E14 | | 15 | 28782449 | | pre-mRNA processing factor 8 [Source:RefSeq peptide;Acc:NP_957270] | |
| 04174E18 | | 25 | 13953753 | | CCR4-NOT transcription complex subunit 1 (CCR4- associated factor 1) [Source:UniProtKB/Swiss- Prot;Acc:A1A5H6] | |
| 01220E39 | | 9 | 15882277 | | MYC binding protein 2 [Source:RefSeq peptide;Acc:NP_001012247] | |
| 75097E9 | | 23 | 28016874 | |  | |
| 77983E1 | | 9 | 5320600 | |  | |
| 55357E2 | | 18 | 39015094 | | RNA polymerase-associated protein LEO1 [Source:UniProtKB/Swiss-Prot;Acc:Q6NYV9] | |
| 15905E10 | | 5 | 9951979 | | leucine-zipper-like transcription regulator 1 [Source:RefSeq peptide;Acc:NP_001074074] | |
| 79949E5 | | 7 | 2831828 | | suppressor of Ty 16 homolog [Source:RefSeq peptide;Acc:NP_001091053] | |
| 13802E2 | | 1 | 845643 | | PCI domain-containing protein 2 (CSN12-like protein) [Source:UniProtKB/Swiss-Prot;Acc:Q5U3P0] | |
| 09285E2 | | 19 | 17733327 | | ribosomal protein L15 [Source:RefSeq peptide;Acc:NP_001003447] | |
| 63694E1 | | 25 | 1300954 | |  | |
| 18049E4 | | 7 | 6277897 | | hypothetical protein LOC570994 [Source:RefSeq peptide;Acc:NP_001098747] | |
| 09549E46 | | 23 | 20847416 | |  | |
| 35956E2 | | 19 | 30520308 | | hypothetical protein LOC553664 [Source:RefSeq peptide;Acc:NP_001018473] | |
